# Adjuvant radiotherapy improves progression-free survival in intracranial atypical meningioma

**DOI:** 10.1186/s13014-019-1368-z

**Published:** 2019-09-02

**Authors:** Saman Moritz Hemmati, Pirus Ghadjar, Arne Grün, Harun Badakhshi, Sebastian Zschaeck, Carolin Senger, Güliz Acker, Martin Misch, Volker Budach, David Kaul

**Affiliations:** 1 Department of Radiation Oncology, Charité Universitätsmedizin Berlin, Berlin, Germany; 2 Department of Radiation Oncology, Ernst von Bergmann Medical Center, Potsdam, Germany; 3 Department of Neurosurgery, Charité Universitätsmedizin Berlin, Berlin, Germany; 4 Klinik für Radioonkologie und Strahlentherapie, Charité Universitätsmedizin Berlin, Augustenburger Platz 1, 13353 Berlin, Germany

**Keywords:** Atypical meningioma, Adjuvant radiotherapy, Fractionated stereotactic radiotherapy

## Abstract

**Background:**

Meningiomas are the most common primary tumors of the central nervous system. In patients with WHO grade I meningiomas no adjuvant therapy is recommended after resection. In case of anaplastic meningiomas (WHO grade III), adjuvant fractionated radiotherapy is generally recommended, regardless of the extent of surgical resection. For atypical meningiomas (WHO grade II) optimal postoperative management has not been clearly defined yet.

**Methods:**

We conducted a retrospective analysis of patients treated for intracranial atypical meningioma at Charité Universitätsmedizin Berlin from March 1999 to October 2018. Considering the individual circumstances (risk of recurrence, anatomical location, etc.), patients were either advised to follow a wait-and-see approach or to undergo adjuvant radiotherapy. Primary endpoint was progression-free survival (PFS).

**Results:**

This analysis included 99 patients with atypical meningioma (WHO grade II). Nineteen patients received adjuvant RT after primary tumor resection (intervention group). The remaining 80 patients did not receive any further adjuvant therapy after surgical resection (control group). Median follow-up was 37 months. Median PFS after primary resection was significantly longer in the intervention group than in the control group (64 m vs. 37 m, *p* = 0.009, HR = 0.204, 95% CI = 0.062–0.668). The influence of adjuvant RT was confirmed in multivariable analysis (*p* = 0.041, HR = 0.192, 95% CI = 0.039–0.932).

**Conclusions:**

Our study adds to the evidence that RT can improve PFS in patients with atypical meningioma.

## Background

Meningiomas are the most common primary tumors of the central nervous system, accounting for about one third of all intracranial tumors. The median age of onset is 65 years [1]. The vast majority of diagnosed meningiomas are benign, with only a small fraction being classified as malignant (grade II and III) [2]. The incidence of meningioma increases with age across both sexes (10 per 100,000 women and 4.4 per 100,000 men) [1]. The increased incidence in women suggests a relationship between hormonal influences and the risk of developing a meningioma. Postmenopausal women receiving hormone replacement therapy have a significantly elevated risk for meningiomas [3].

The histological origin of meningiomas is based on clonal proliferation of arachnothelial cells of the meninges [4]. Different genetic causes are accountable for the development and progression of sporadic and familial meningiomas [5]. Sporadic meningiomas are mainly associated with focal chromosomal deletion and inactivation of neurofibromatosis type 2 (*NF2*) tumor suppressor gene on chromosome 22, which encodes a cytoskeletal-associated protein with an inhibitory effect on the cell cycle [6]. Familial meningiomas are related to various mutations in the *NF2* gene [7, 8].

The fourth edition of the WHO classification of tumors of the central nervous system (2007) characterized meningiomas into grades I, II, and III [9]. This classification was primarily based on histopathological criteria: Grade I (benign) was characterized by low proliferation rates and a lack of anaplastic features; grade II (atypical) by elevated mitotic rates and necrosis [9, 10]; grade III (anaplastic) was defined by nuclear atypia and a highly enhanced mitotic activity [10]. The fifth and most recent edition of the WHO classification (2016) added the criterion of infiltrating growth for the diagnosis of atypical meningioma (WHO grade II) [11].

In patients with newly diagnosed meningioma, a wait-and-see approach is considered when clinical conditions allow it, with periodic clinical and radiographic follow-up. However, regardless of the WHO grade, surgical removal is still considered the treatment of choice [12]. Resection is indicated for large tumors, symptomatic patients, or fast growing tumors with mass effect [13]. The extent of neurosurgical resection depends on tumor localization, and is measured using the Simpson grade, which is based on the surgeon’s intraoperative assessment [14]. The operative intervention aims at complete removal of all tumor parts, including associated dura and underlying bone (Simpson grade I), while preserving neurological function [13]. However, this may not always be possible due to infiltration of venous sinuses, adherence to blood vessels and cranial nerves or localization at the base of the dura were dura cannot simply be removed.

Modern radiation therapy (RT) is becoming increasingly important in the treatment of meningiomas. RT can be performed as stereotactic radiosurgery (SRS) or as fractionated stereotactic RT (FSRT). Patients with WHO grade I meningiomas, who are not undergoing surgery, benefit from primary RT. In patients with incompletely resected WHO grade I meningiomas, adjuvant RT provides longer progression-free survival (PFS), but a wait-and-see approach is preferred due to the slow growth rate of the tumors [12, 15, 16]. In case of anaplastic meningiomas (WHO grade III), adjuvant fractionated RT is generally recommended, regardless of the extent of surgical resection [12]. This has been shown to improve local tumor control and recurrence rates [17, 18]. For atypical meningiomas (WHO grade II), the primary aim is to achieve a radical surgical tumor extirpation [12]. Despite elevated recurrence rates, optimal postoperative management for atypical meningioma has not been clearly defined yet. In particular, the role of adjuvant fractionated RT has remained controversial. Depending on the extent of surgical resection, various retrospective studies showed lower recurrence rates and improved overall survival (OS) for adjuvant irradiated WHO grade II meningiomas [19–21]. However, a large number of studies have found no definite advantage of adjuvant RT, emphasizing the prognostic significance of surgical resection and the risk of radiation-induced toxicity [22–25]. Thus, retrospective studies regarding adjuvant RT in atypical meningiomas have demonstrated inconsistent results, and the results of ongoing randomized-controlled trials are not yet available [26, 27].

The aims of this retrospective study were to investigate the role of adjuvant RT in WHO grade II meningiomas and to identify prognostic factors that have an impact on PFS and OS.

## Methods

### Study population, data collection, and course of treatment

We conducted a retrospective analysis of 99 patients treated for intracranial atypical meningioma at Charité Universitätsmedizin Berlin from March 1999 to October 2018. Patient identification was based on the review of the hospital’s clinical cancer registry. Only adult patients who underwent primary tumor resection were included. The postoperative treatment decisions were made by an interdisciplinary tumor board. Considering the individual circumstances (risk of recurrence, anatomical location, etc.), patients were either advised to follow a wait-and-see approach or to undergo adjuvant RT. The regional ethics committee approved this study (EA2/094/18).

### Technical equipment and treatment planning

From 1995 to 2003, meningioma patients underwent “sharp” fixation using a stereotactic head ring and an oral bite plate. A 6-MV linear accelerator (LINAC) (Varian Medical Systems, USA) with an add-on micro-multileaf collimator (BrainLAB, Germany) was used. Coordinates for SRS were set by a laser-based stereotactic localizer, which allowed the delivery of shaped beams. In 2004, the department started using Novalis Tx with beam shaping capability using built-in multileaf collimator (MLC) and image guidance with ExacTrac (Varian Medical Systems, USA, and BrainLAB, Germany). The image-guided frameless system enabled imaging with high accuracy independent of couch position. A three-dimensional treatment planning based on CT-co-registered with MRI was calculated using BrainScan (BrainLAB, Germany), which was later replaced by iPlan RT (BrainLAB, Germany). The gross tumor volume (GTV) was defined as the area of contrast enhancement on T1-weighted MR images, and the planning target volume (PTV) included a 1–2 mm isotropic safety margin. The dose was prescribed to a reference point, representing 100%. Patients received 95% of the prescribed dose at the PTV margin.

### Endpoints and variables

Follow-up was defined as the period between primary tumor resection and last patient contact or death. PFS was the primary endpoint of this study. OS was a secondary endpoint. Twelve intracranial tumor locations were distinguished and divided into three groups depending on their surgical risk according to the CLASS algorithmic scale [28].

### Statistics

Statistical analysis was performed using SPSS Statistics (v. 25.0, IBM Inc., USA). Kaplan-Meier survival analysis was used to calculate PFS after primary resection and OS. The prognostic value of variables was evaluated using univariable Cox-regression analysis. A multivariable Cox-regression analysis was performed to exclude possible confounding factors. The frequency distribution of continuous variables (patient age at first diagnosis and pretherapeutic tumor volume) was examined for significant differences with the unpaired Student’s *t* test. A chi-square test was used to compare the distribution of nominal variables (sex, intracranial tumor localization, and type of salvage therapy). The distribution of ordinal variables (Simpson resection grade) was compared using the Mann-Whitney U test. For all statistical analyses, data were considered statistically significant for *p* ≤ 0.05.

## Results

### Patient characteristics

This analysis included 99 patients with atypical meningioma (WHO grade II). Nineteen patients received adjuvant RT after primary tumor resection (intervention group). The remaining 80 patients did not receive any further adjuvant therapy after surgical resection (control group). Patient characteristics are shown in Table 1. The two groups were well balanced in terms of age, tumor volume, sex, tumor location and Simpson grade. Median follow-up until last contact or death was 37 months. Most patients in the intervention group received a single dose of 1.8 Gy to a total dose of 54 or 59.4 Gy (median total dose was 59.4 Gy).

**Table 1:** Patient characteristics. SRS = stereotactic radiosurgery

Characteristics	Total *n* = 99	Intervention *n* = 19	Control *n* = 80	*p*-value
	Median (Min/Max)	Median (Min/Max)	Median (Min/Max)	
Patient age at first diagnosis (years)	59	59	59	0.49
	(22/84)	(26/75)	(22/84)	
Pretherapeutic tumor volume (cm^3^)	29.7	47.1	26.1	0.16
	(0.8/153.9)	(12.6/90.2)	(0.8/153.9)	
	n (%)	n (%)	n (%)	
Sex				
Male	42 (42.4)	8 (42.1)	34 (42.5)	0.98
Female	57 (57.6)	11 (57.9)	46 (57.5)	
Tumor location				
Convexity	36 (36.4)	7 (36.8)	29 (36.3)	0.91
Olfactory groove / Planum sphenoidale	4 (4.0)	0 (0.0)	4 (5)	
Lateral sphenoid wing / Temporal bone	6 (6.1)	2 (10.5)	4 (5)	
Parasagittal / Falx	23 (23.2)	4 (21.1)	19 (23.8)	
Cerebellopontine angle	3 (3.0)	1 (5.3)	2 (2.5)	
Petroclival	1 (1.0)	0 (0)	1 (1.3)	
Medial sphenoid wing / Clinoid / Orbital	17 (17.2)	4 (21.1)	13 (16.3)	
Tuberculum sellae	3 (3.0)	0 (0)	3 (3.8)	
Cavernous sinus	0 (0.0)	0 (0)	0 (0)	
Tentorial	6 (6.1)	1 (5.3)	5 (6.3)	
Foramen magnum	0 (0.0)	0 (0)	0 (0)	
Optic nerve sheath	0 (0.0)	0 (0)	0 (0)	
Simpson grade				
1	24 (24.2)	5 (26.3)	19 (23.8)	0.39
2	35 (35.4)	4 (21.1)	31 (38.8)	
3	1 (1.0)	0 (0)	1 (1.3)	
4	11 (11.1)	4 (21.1)	7 (8.8)	
5	1 (1.0)	1 (5)	0 (0)	
Not documented	27 (27.3)	5 (26.3)	22 (27.5)	
Salvage therapies				
No recurrence	58 (58.6)	16 (84.2)	42 (52.5)	
Resection	3 (3.0)	0 (0.0)	3 (3.8)	
Radiotherapy	19 (19.2)	2 (10.5)	17 (21.3)	
Resection and Radiotherapy	19 (19.2)	1 (5.3)	18 (22.5)	
Fractionation scheme				
Not irradiated	80 (80.8)	0 (0)	80 (100)	
Normofractionation (1.8–2.2 Gy)	17 (17.1)	17 (89.5)	0 (0)	
Hypofractionation (> 2.2–5 Gy)	1 (1)	1 (5.3)	0 (0)	
SRS (> 5 Gy)	1 (1)	1 (5.3)	0 (0)	

### Progression-free survival

**Fig. 1:**
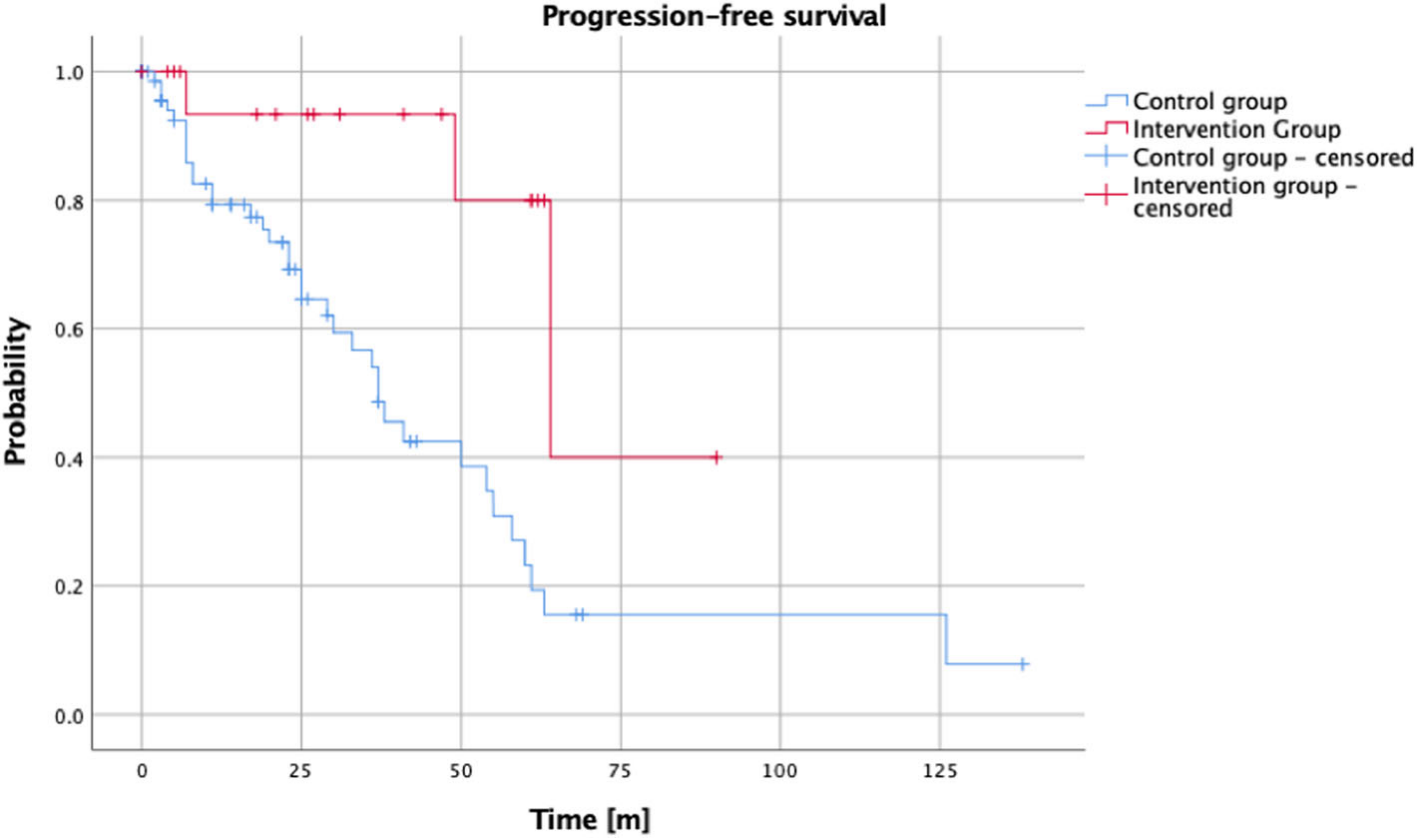
Cumulative progression-free survival after primary resection for intervention group (*n* = 19) and control group (*n* = 80) respectively (*p* = 0.009, HR = 0.204, 95% CI = 0.062–0.668)

Median PFS after primary resection was significantly longer in the intervention group than in the control group (64 m vs. 37 m, *p* = 0.009, HR = 0.204, 95% CI = 0.062–0.668, Fig. 1). The influence of adjuvant RT was confirmed in multivariable analysis (*p* = 0.041, HR = 0.192, 95% CI = 0.039–0.932, Table 2). The factor “sex” showed a significant impact on PFS in univariable analysis (*p* = 0.014, HR = 0.441 95% CI = 0.229–0.850). However, this was not confirmed in multivariable analysis. Simpson resection status was identified as a factor influencing PFS in univariable analysis (*p* = 0.009, HR = 1.488, 95% CI = 1.106–2.001). In multivariable analysis, a significant role of Simpson resection status was confirmed (*p* = 0.032, HR = 1.655, 95% CI = 1.043–2.626). Patient age, tumor volume, and tumor location (skull base vs. other, and high-risk location according to the CLASS algorithmic scale vs. other) did not show a significant influence on PFS.

**Table 2:** Uni- and multivariable analyses of progression-free survival. *P*-values ≤0.05 were defined as statistically significant (*). Tumor localization in multivariable analysis was performed according to the CLASS algorithm (and not as “skullbase vs non-skullbase”) because this information is already included in the CLASS algorithm

	Univariable			Multivariable		
	*p*	HR	95% CI	*p*	HR	95% CI
Adjuvant radiotherapy (Intervention vs. control)	0.009^*^	0.204	0.062–0.668	0.041^*^	0.192	0.039–0.932
Sex (Male vs. female)	0.014^*^	0.441	0.229–0.850	0.422	0.666	0.247–1.796
Simpson resection grade^14^ (Grade IV/V vs. Grade I/II/III)	0.009^*^	1.488	1.106–2.001	0.032^*^	1.655	1.043–2.626
Patient age at first diagnosis (> median vs. ≤ median)	0.465	1.278	0.662–2.468	0.750	1.175	0.436–3.163
Pretherapeutic tumor volume (> median vs. ≤ median)	0.908	0.96	0.478–1.928	0.773	1.181	0.380–3.669
Intracranial tumor localization (CLASS moderate/high risk vs. low risk)	0.301	1.184	0.860–1.630	0.614	1.163	0.647–2.093
Intracranial tumor localization (non-skull base vs. skull base)	0.5	0.998	0.991–1.004			

### Overall survival

Univariable analysis of potential factors influencing OS is shown in Table 3. Higher age (>median) was identified as a significant predictor of OS in univariable analysis. However, this was not confirmed in multivariable analysis.

**Table 3:** Univariable analysis of factors influencing overall survival after primary resection of atypical meningioma. *P*-values ≤0.05 were defined as statistically significant (*). Tumor localization in multivariable analysis was performed according to the CLASS algorithm (and not as “skullbase vs non-skullbase”) because this information is already included in the CLASS algorithm

	Univariable			Multivariable		
	*p*	HR	95% CI	*p*	HR	95% CI
Adjuvant radiotherapy (Intervention vs. control)	0.661	0.709	0.152–3.301	0.309	0.238	0.015–3.797
Sex (Male vs. female)	0.49	0.654	0.196–2.185	0.954	1.052	0.191–5.781
Simpson resection grade^14^ (Grade IV/V vs. Grade I/II/III)	0.575	1.172	0.674–2.037	0.295	1.452	0.722–2.919
Patient age at first diagnosis (> median vs. ≤ median)	0.048*	3.978	1.012–15.641	0.095	5.278	0.748–37.243
Pretherapeutic tumor volume (> median vs. ≤ median)	0.143	3.28	0.668–16.104	0.678	1.439	0.258–8.04
Intracranial tumor localization (CLASS moderate/high risk vs. low risk)	0.711	0.893	0.492–1.621	0.463	0.691	0.258–1.852
Intracranial tumor localization (non-skull base vs. skull base)	0.723	1.002	0.990–1.015			

## Discussion

In this retrospective study, we investigated the influence of adjuvant RT on PFS in atypical meningioma patients. In the multivariable analysis, patients who received adjuvant RT demonstrated significantly longer PFS rates compared with patients who did not receive adjuvant RT. The second significant factor in multivariable analysis of PFS was Simpson grade of resection.

In the present study, male patients showed worse PFS rates in univariable analysis. However, the factor gender was eliminated in our multivariable model for PFS. This is in accordance with most studies, which have not found a significant association between gender and PFS in grade II meningioma [29–32]. Nonetheless, two published studies have shown an association between females and tumor relapse in univariable analysis [33, 34].

The median age in our patient cohort was 59 years, which is comparable with the median age reported in other studies [19, 32, 35]. We did not find an association between age and PFS in uni- and multivariable analyses. Higher age was a significant predictor of worse OS in univariable analysis. Our finding, that age is not a predictor of PFS, is in accordance with most published data. However, some studies have linked younger patient age to improved PFS or OS. Champeaux and colleagues (2016) showed that patients younger than 57 years at surgery had a significantly higher PFS rate [36]; Endo and colleagues (2016) demonstrated that age > 60 years correlated with low PFS and OS [29].

In this study adjuvant RT significantly improved PFS rates in uni- and multivariable analyses. However, the PFS benefit did not translate into an OS benefit. The fact that the benefit of adjuvant RT for PFS did not translate into an OS benefit might be explained by the high rate of salvage RT: Of 38 patients who showed recurrence in the control group 35 received salvage RT at some point. The PFS benefit is in accordance with numerous other studies [19, 21, 32], and only one study showed reduced PFS rates in patients who received adjuvant RT [37].

A positive correlation between PFS and Simpson grade I-III vs. Simpson grade IV-V was shown in univariable and multivariable analyses, that did not translate into an OS benefit. Most studies have shown a positive correlation between the extent of surgical resection and PFS in grade II meningioma [21, 22, 24, 30, 35]. However, it should be mentioned that some studies were not able to confirm the described correlation [32, 37].

We did not find any significant influence of tumor location on PFS or OS when looking at the CLASS algorithmic scale (low risk vs. moderate and high risk), and when looking at skull base tumors vs. other locations. Nonetheless, some studies have found a higher risk of recurrence in “deep locations”, like the skull base and the cerebral ventricles [32]. These results might have been confounded, by the fact that “deep locations” are more difficult to resect, causing a higher rate of subtotal resections. Contrary to the mentioned study, Vranic and colleagues showed reduced PFS rates in patients with tumors in the parasagittal/falcine region [38].

We did not find any correlation between tumor volume and PFS or OS. Other studies have shown low recurrence-free survival rates for large diameter tumors [34].

The present study has some limitations. Firstly, the retrospective approach is prone to bias. Secondly, it should be pointed out that median follow-up to last contact or death was only 37 months. Patient numbers are relatively small in the intervention group. Additionally, a high rate of not-documented Simpson grades may interfere with interpretation of the results of this study. Other factors than Simpson grade may better predict tumor control rate, e.g. TERT mutation status in the tumor, which was not assessed in this study.

## Conclusions

Grade II meningiomas show a high tendency of tumor recurrence. Standard treatment of grade II meningiomas is surgical resection, with the role of adjuvant RT being unclear. In the present study, Simpson grade I-III resection and adjuvant RT improved PFS in multivariable analysis. Thus, our study adds to the evidence that RT can improve PFS in patients with atypical meningioma.

## Data Availability

Data in the manuscript are available by contacting the corresponding author.

## Abbreviations

CI: Confidence intervall
FSRT: Fractionated stereotactic radiotherapy
GTV: Gross tumor volume
HR: Hazard ratio
LINAC: Linear accelerator
MLC: Multileaf collimator
NF2: Neurofibromatosis type 2
OS: Overall survival
PFS: Progression-free survival
PTV: Planning target volume
RT: Radiotherapy
SRS: Stereotactic radiosurgery
WHO: World health organization
